# Mathematics is different: student and tutor perspectives from Ireland and Australia on online support during COVID-19

**DOI:** 10.1093/teamat/hrab014

**Published:** 2021-09-27

**Authors:** Claire Mullen, Jim Pettigrew, Anthony Cronin, Leanne Rylands, Donald Shearman

**Affiliations:** 1 School of Mathematics and Statistics, University College Dublin, Belfield, Dublin 4, D04 V1W8, Ireland; 2 Mathematics Education Support Hub, Western Sydney University, Locked Bag 1797, NSW 2751, Penrith; 3 Centre for Research in Mathematics and Data Science, and Mathematics Education Support Hub, Western Sydney University, Locked Bag 1797, NSW 2751, Penrith

## Abstract

From March 2020, the Mathematics Support Centre at University College Dublin, Ireland, and the Mathematics Education Support Hub at Western Sydney University, Australia, moved wholly online and have largely remained so to the point of writing (August 2021). The dramatic and swift changes brought on by COVID-19, in particular to fully online modes of teaching and learning including mathematics and statistics support (MSS), have presented students and tutors with a host of new opportunities for thinking and working. This study aims to gain insight both from students and tutors about their experience of wholly online learning and tutoring in the COVID-19 era. In this sense, it represents a ‘perspectives’ study, the idea being that before we examine specific aspects of this experience, it would be best to know what the issues are. Employing a qualitative analysis framework of 23 one-on-one interview transcripts with tutors and students from both institutions in Australia and Ireland, we identified five key themes as central to the shared experiences and perspectives of tutors and students. In this study, we discuss three of these themes in relation to the new normal with the intention of supporting MSS practitioners, researchers and students going forward. The themes describe the usage of online support, how mathematics is different and the future of online MSS.

## 1 Introduction

Mathematics and statistics support (MSS) is complementary to regular timetabled teaching activities such as formal lectures, tutorials, problem solving classes and laboratories. In many countries, MSS has come about as a response to what is commonly known as the ‘mathematics problem’. This refers to a complex combination of issues including the under preparedness of incoming undergraduate students from secondary school, the growing diversity in mathematical backgrounds of students due to widening participation agendas and the ever-increasing requirement of employers for graduates to have quantitative reasoning skills. Extensive literature reviews of MSS, including the evolving ‘mathematics problem’ spanning over 25 years of scholarship, have been conducted by Matthews *et al.* (2013) and Lawson *et al.* (2020), with country specific reports of the problem and MSS in Australia (Barrington & Brown, 2014; Productivity Commission, 2019), Ireland (Gill *et al.*, 2010; MacCraith, 2016), the UK (Grove *et al.*, 2020), Germany (Schürmann *et al.*, 2021) and the USA (Mills *et al.*, 2020), among other nations.

While in-person MSS provision has become the norm, its online equivalent has seen slower take up in the on-campus traditional model of higher education. The technology to provide MSS online has existed for some time but it was not until March 2020 that the COVID-19 pandemic forced staff and students to vacate campuses and to embrace fully online learning and teaching. Prior to this, there had been limited research investigating the online offering of MSS (Cronin & Breen, 2015; Mac an Bhaird *et al.*, 2021; Pettigrew & Shearman, 2013), and until recently the consensus from both students and tutors was that online MSS cannot replace the quality experience of the in-person context. Now that online MSS has been experienced by many for a considerable amount of time, there is significant interest in the evaluation of such support, partly to inform decisions about its use in the future. With this in mind, this paper aims to address the following research question:

What are the issues common to an Australian and an Irish university, from both the student and tutor perspective during the COVID-19 era, pertaining to the use and future of online MSS?

## 2 Literature review

### 2.1 MSS in Australia and Ireland

In Australia, MSS goes back at least as far as 1973, with dedicated centres in universities created to provide MSS appearing in, and possibly predating, 1984 (Dzator & Dzator, 2018; MacGillivray, 2009). By 2007, some form of MSS was provided by 32 of Australia’s 39 universities (MacGillivray, 2009). There are a variety of documented ways in which MSS can benefit students, for example, better grades, increased confidence and greater retention. In Australia, there are many examples from the literature reporting on such benefits to students from MSS engagement including Rylands & Shearman (2018), Jackson (2013), Jackson & Johnson (2020), Hillock & Khan (2019), MacGillivray (2009) and Dzator & Dzator (2018).

MSS was established as early as 1999 in the Republic of Ireland (Cronin *et al.*, 2016) with the first support centre set up in 2001 at the University of Limerick. As of 2016, there was MSS of some description in almost all higher education institutions on the island of Ireland (Cronin *et al.*, 2016). Again, these services were established in response to variants of the ‘mathematics problem’ mentioned earlier. In Ireland, there have been articles published on the positive impact of MSS: on students’ grades (Jacob & Ní Fhloinn, 2018), student retention (O’Sullivan *et al.*, 2014) and teaching practice (Cronin *et al.*, 2019; Cronin & Meehan, 2020).

### 2.2 Online mathematics support

A survey of MSS in Ireland up to 2015 (Cronin *et al.*, 2016) found that of 30 institutions, 25 offered MSS. Only 12 of these offered some online support (here online included advertising and links to resources) and only one offered synchronous interactions with a tutor. A 2018 UK and Ireland survey (Mac an Bhaird *et al.*, 2021) of institutions’ online MSS presence received a response rate of approximately 28% from institutions known to have provided MSS at that time. Some 20 of 33 respondents stated that they had offered synchronous MSS sessions. In general, this virtual support was not taken up by students regularly and the technology was rated poorly in terms of student learning, synchronisation and wi-fi connectivity. Barriers to providing an online MSS presence included staffing issues, technology, funding, student awareness of ICT and preference (practitioners) for face-to-face tuition.

There were a number of surveys, both of MSS practitioners (Hodds, 2020; Johns & Mills, 2021) and undergraduate mathematics students (Meehan & Howard, 2020), conducted within the initial months of the pandemic-enforced changes to higher education in mid 2020. Hodds (2020) describes the differences in pre- and post-pandemic MSS offerings from 78 higher education institutions around the world including 19 outside the UK. This report details a significant decrease in MSS student engagement when the move to fully online MSS occurred. Johns & Mills (2021) discuss best practice for online MSS and offer recommendations for practitioners based on the views of 28 MSS leaders in the USA. The undergraduate student perspective of the affordances and constraints of online mathematics learning during the initial COVID period, in addition to a set of recommendations for lecturers going forward, is reported in Meehan & Howard (2020).

### 2.3 Online teaching and learning

While there has been little research reported on online MSS, there is a large body of research into online learning, some of which includes online mathematics. It is now timely, with students being forced to study online, to consider the body of literature about online learning in order to improve online MSS.

Since the emergence of online education in the early to mid 1990s, a vast body of research has arisen to examine its effectiveness and explore its potential (Martin *et al.*, 2020). Separating the studies that address broad questions of the value and purpose of online education from those that seek to unearth nuanced, contextual and discipline-specific issues is an important, though infrequently considered, task (Paechter & Maier, 2010; Protopsaltis & Baumi, 2019; Smith *et al.*, 2008; Trenholm, 2013; Trenholm *et al.*, 2019). This has particular implications for research whose aim is to investigate online teaching and learning approaches in mathematics, a discipline that is special across a range of dimensions, including pedagogy, learning psychology, use of abstraction, symbolic language, idiomatic written and notational conventions and application of ‘sequentially-acquired’ conceptual knowledge (Smith *et al.*, 2008; Trenholm *et al.*, 2019).

Much attention is given in the literature to the benefits of online education, for example, flexibility, personalization, convenience, expanded access, time efficiency, affordance of anonymity, increased learning environment amenity and protection from distractions. However, critical appraisals of its capacity to deliver high-quality learning are rare (Danielson *et al.*, 2014; Figlio, 2016; Jaggars, 2014; Protopsaltis & Baumi, 2019; Trenholm, 2013; Trenholm *et al.*, 2016). This is regrettable as the distinction has special relevance to the debate about the comparative effectiveness of online and face-to-face approaches to teaching and learning. Moreover, the debate is complicated by evidence that the advantages and academic rewards of online learning vary depending on a variety of factors. For example, socio-economically disadvantaged students suffer substandard learning outcomes when studying online. They are also ill-equipped to motivate themselves, regulate, organize, structure or direct their own online learning and struggle with time management and developing (or activating) independent learning skills (same references as before) (Barshay, 2015; Cavanaugh & Jacquemin, 2015; Otter *et al.*, 2013; Protopsaltis & Baumi, 2019; Xu & Jaggars, 2013, 2014).

The challenges facing teachers working in online environments, many of whom have been forced due to COVID-19 to negotiate exclusively digitally mediated relationships with their students, are profound. Some researchers suggest that, no matter what the medium, pedagogy is preeminent (Bernard *et al.*, 2004; Meehan & Howard, 2020; O’Neill *et al.*, 2004). Learning frameworks and guiding pedagogical principles inform tutors’ practice and these need to be adjusted to accommodate online relations with students. Concerns about how to recapture the immediacy of ‘short-cycle’ interactions, the ‘magic of a good face-to-face tutorial’ and other behavioural phenomena that are commonly found in ‘co-present’, face-to-face communication (such as nonverbal cues and continuous turn-taking) test the skills of tutors (Bork & Rucks-Ahidiana, 2013; Lowe *et al.*, 2016; Trenholm, 2013; Trenholm *et al.*, 2016). In addition to this is the array of technological constraints and affordances that teachers must incorporate into their practice, some of which are doubly demanding for mathematics teachers struggling to overcome the dominance in digital classrooms of ‘qwerty- and mouse-based communication’ and ‘rigid syntax constraints’ (Trenholm *et al.*, 2019).

A strong refrain in studies that examine issues affecting online mathematics education is that ‘mathematics is different’ (Smith *et al.*, 2008; Trenholm, 2013; Trenholm *et al.*, 2019). Here, it is claimed that the various digital platforms that are used for communicating (in written, oral and non-verbal forms) and sharing work with students can flatten the learning environment and make it more difficult for teachers to ply their skill (Meehan & Howard, 2020). It is perhaps not surprising, given the high levels of abstraction and perceived ‘hardness’ of mathematics that students claim they ‘cannot teach [the subject] to themselves’ and expect their learning to be sustained by fulsome instructional guidance (this contrasts with the situation for some humanities subjects, for example, which are perceived as ‘soft’) (Jaggars, 2014; Trenholm, 2013). Standard mathematical instructional techniques, such as instructor modeling of problem solving, animated use of visual-spatial components in diagrams and demonstrations and timely application of corrective feedback, have to be reimagined for online use. This challenges teachers to develop new modes of practice (Smith *et al.*, 2008). A possible consequence of these discipline-specific issues is students’ stated preference for studying mathematics, above other disciplines, face to face (Jaggars, 2014; Xu & Jaggars, 2014).

A systemic review of research on online teaching and learning from 2009 to 2018 found that one of the least studied themes was institutional support (Martin *et al.*, 2020). This contrasts with the amount of research attention devoted to the problem of student retention in an online context (Boles *et al.*, 2010; Sorensen & Donovan, 2017; Trenholm *et al.*, 2019) and issues surrounding transition more broadly (Briggs *et al.*, 2012; Hernandez-Martinez *et al.*, 2011). Institutional support of mathematics learning can take many forms but is generally seen as the sum of a set of enabling services whose purpose is to improve students’ academic performance, bolster their confidence, instil within them a sense of solidarity and community and dispose them positively towards a discipline that is often perceived to be impenetrably difficult or the source of significant anxiety (Heyman, 2010; Ludwig-Hardman & Dunlap, 2003). More research is required to investigate questions of tutors’ and students’ perceptions and preferences related to their provision or use of online MSS as this genre is only in its early stages of development. Research that is qualitative and sensitive to the challenges and opportunities presented by COVID-19 is even less prevalent but no less important.

## 3 Background

Western Sydney University (WSU) is a multi-campus university in the western part of Sydney, Australia. In 2019, the university had approximately 50,000 students of which 79% were undergraduate students. WSU is a genuine multi-campus university in the sense that there is no main campus, and many services and degree programs are available on some or all of the campuses. WSU currently does not list secondary school mathematics as a prerequisite for any degrees.

WSU has had mathematics support staff for over 25 years, although with very few staff in the early years. For example, in 2000, there were the equivalent of 1.5 full-time MSS staff. In 2011, the Mathematics Education Support Hub (MESH) was created to provide MSS to students and MESH currently has the equivalent of just over five full-time staff.

MESH provides support for all students, except for research degree students who need assistance with statistical analyses. Support is usually provided face to face on six campuses as well as an online answer service (a discussion board where staff respond to posts). There is no physical space run by MESH; drop-in MSS is offered at advertised times in various campus libraries and teaching spaces are booked for other MSS activities.

The face-to-face services provided by MESH pre-COVID-19 included drop-in support, test and examination preparation workshops for many first-year subjects,^1^ workshops run during the 4 weeks before new students begin their formal studies and workshops for particular disciplines (e.g., nursing).

On 18 March 2020, which was Week 3 of a 15-week semester, all face-to-face MESH MSS moved online using Zoom. The drop-in support which had run on six campuses continued to be drop-in support, mostly using audio with students’ videos off (by students’ choice). Workshops also ran via Zoom, using breakout rooms with mixed use of video, audio and chat. Support remained online for the rest of the year. During the break between Autumn and Spring MESH ran some discipline specific online workshops. In summary, and for the purposes of this study, MESH provided wholly online tutoring/learning for all students for 28 weeks, which for the students in this study was just three weeks short of their full academic year.

University College Dublin (UCD), Ireland, is a research-intensive university currently ranked within the top 1% of higher education institutions world-wide. In 2019/20, UCD had over 32,000 registered students of which 67% were undergraduates and 29% international students. It is consistently the university of first choice among school leavers in Ireland.

The UCD Mathematics Support Centre (MSC) was established in 2004. It is staffed by a full-time manager and approximately 20 tutors are hired each year. These are predominantly graduate students with a handful of undergraduates hired as peer mentors also. Annually, the MSC supports in excess of 5,500 student visits from over 250 distinct subjects across all six colleges of the university. Since October 2015, only students registered to preparatory, first- or second-year subjects may access the MSC. Up to March 2020, apart from a short pilot using Slack.com during peak demand of final examinations, the MSC did not offer synchronous online MSS.

The MSC started providing wholly online MSS from 23 March 2020, Week 8 of the 12-week teaching semester, (January to May) of 2019/20. This was conducted through the institution’s virtual learning environment using virtual classroom video conferencing software. All sessions were appointment based with students booking 30 minute slots. Typical sessions were conducted with both tutors’ and students’ cameras off thus relying on audio and/or chat. The MSC was fully online for first semester 2020/21 (September to December) when MSS ran from Weeks 3 to 12. In contrast to MESH which had 28 weeks of wholly online MSS the MSC provided MSS wholly online for a total of 14 weeks during the COVID-19 period of this study.

The initial move to online MSS saw a dramatic drop in the number of users at both MSC and MESH. From March to May 2020, the end of the second semester, the MSC service experienced a 79% drop in usage on the same period in the previous year. This amounted to 245 visits from 85 distinct students compared to the corresponding figures of 1,149 visits from 412 distinct students in 2019. This was despite the online MSS service remaining open for three extra weeks—the examination period was extended due to COVID-19—which had not been done in previous years.

The MESH drop-in service saw 604 students from April to December 2020, whereas from April to December 2019 there were 1,156 students (a drop of 46%). MESH workshops, almost all of which are held after March, attracted 2,545 students in 2019 and 1,971 in 2020, a drop of 23% (a student is counted each time they use a MESH service).

## 4 Method

Data were collected in the form of transcripts emanating from 23 one-on-one Zoom interviews conducted by the lead author from late October to late November 2020. Interviews were conducted with seven WSU students (AS1–AS7), four WSU MESH tutors (AT1–AT4), six UCD students (IS1–IS6) and six UCD MSC tutors (IT1–IT6). The semi-structured interviews ranged in length from 14 to 44 minutes with a mean length of 28 minutes. The interviewer used a single set of questions for both UCD and WSU appropriately adapted for the student/tutor context—the interview questions are available in Appendix A. The interview questions were designed based on the authors’ extensive experience as mathematics educators and MSS researchers and previous research highlighted in Section 2 and came about through multiple discussions about MSS in both universities pre- and during the pandemic. The questions were piloted to ensure that they were open enough to allow for rich responses but restricted enough to target the research question.

We provide a comprehensive description of the coding process used for the current study, with the aim that it will act as a template for MSS practitioners and researchers interested in adopting this, or a similar, approach.

### 4.1 Qualitative analysis and coding process

Thematic, deductive, semantic coding, as defined by Braun & Clarke (2006), was used to analyse the interview transcripts. Segments of text that shared a theme in common were labelled (coding), the codes were created based only on the data, not with any theory or expected themes in mind (deductive), and finally, the codes/themes were based on the explicit meaning of the interview text (semantic).

**Fig. 1.**
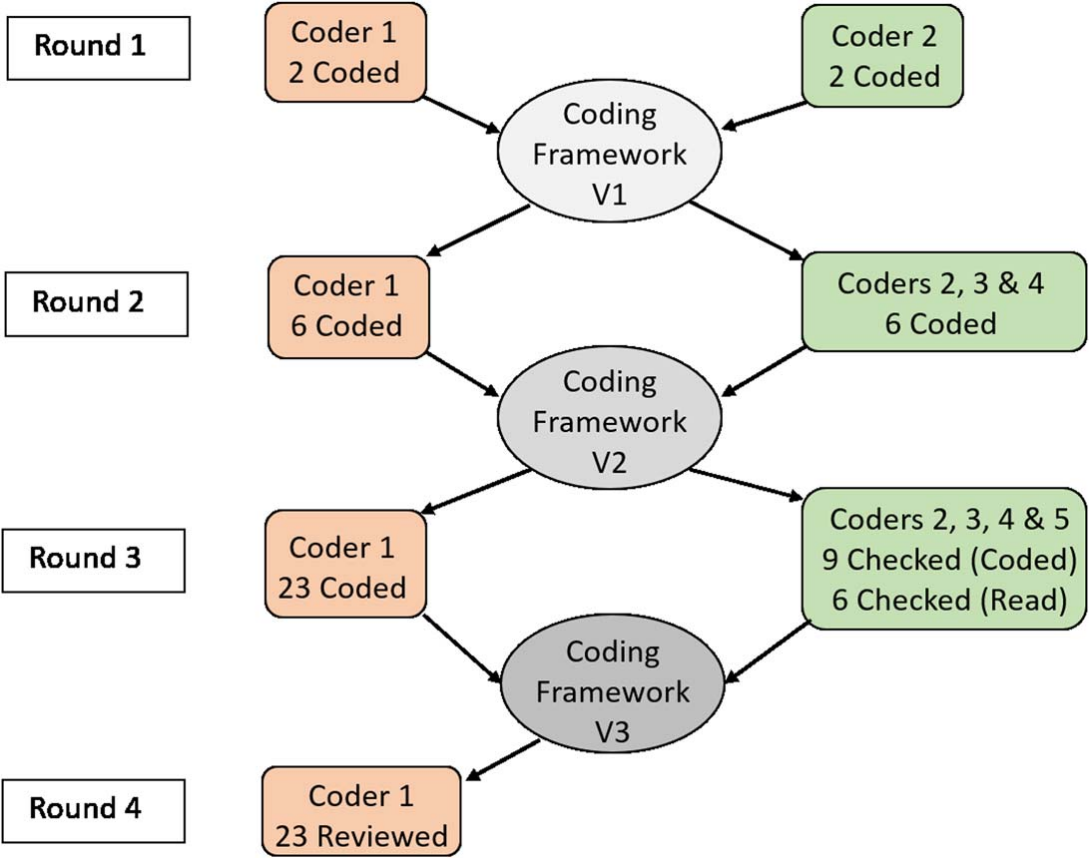
Creation of the coding framework over four rounds of coding with five coders.

The coding framework was built over four rounds of coding as outlined below and shown in Fig. 1. Throughout these rounds the primary coder the lead author was assisted by the co-authors in coding to ensure the following: (a) the coding framework was as expansive and well defined as possible; (b) the coding framework was used consistently throughout all 23 interviews; (c) the context of the WSU tutors’ and students’ interviews was not misunderstood by the UCD-based first author. Through this multi-round, multi-coder process a strong coding framework with consistently coded interviews was created to aid the theme development and identification process that constituted the final step in the analysis. The qualitative analysis software program MAXQDA was used for coding in Rounds 3 and 4, with Microsoft Word and Excel used for Rounds 1 and 2.

#### 4.1.1 Round 1: initial coding framework development ($n=2$ interviews coded)

To build the initial coding framework, the lead and third author both independently coded two interviews, a WSU student and a UCD tutor, in accordance with the method outlined by Thomas (2006). This coding was in vitro, meaning segments of the interviews were highlighted and given concise descriptive titles (for example Issues Wi-fi). The authors then exchanged their initial coding frameworks, and the list of codes and their definitions that each author used independently in analysing the interviews. They then independently compared their coding frameworks and subsequently met to discuss this and merge the two initial frameworks into one. The two frameworks overlapped significantly and version one of the coding framework, V1, was created.

#### 4.1.2 Round 2: framework clarification and expansion ($n=6$ interviews coded)

To clarify and expand the coding framework, six further interviews were coded using version one with the intention to add more codes where necessary and develop the definitions of the existing codes. This ensured that the codes were understandable and usable and that as many additional codes as necessary were added to the framework before coding the majority of interviews. The lead author coded all six of these interviews and the second, third and fourth authors each coded two or three interviews. Again, this process was conducted independently. Two WSU tutors, two UCD students, one WSU student and one UCD tutor were coded in this round so that version two of the framework would be built upon a representative sample of all four interviewee groups.

The lead author received the coded transcripts from the rest of the team and after comparing each coded interview with their own coded transcripts, organized all codes used in Rounds 1 and 2 with their definitions. The codes created in Round 2 were compared to the codes in version one and were merged with another code where appropriate or added to the framework to create version two, V2, of the coding framework.

#### 4.1.3 Round 3: coding the data set with consistency checks. ($n=23$ interviews coded)

Version two of the coding framework was used for the first round of coding on the other 14 interviews that were not coded in Rounds 1 or 2 and to recode the nine interviews already coded in Rounds 1 and 2 with version two of the codes. This was completed by the lead author; however, checks for consistency utilizing Thomas’s ‘Check on the clarity of categories’ were completed by the other authors (Thomas, 2006). The lead author sent the segments of text from an interview that they had coded to a fellow researcher, without the codes attached, and they coded the text segments to ensure reliable use of the coding framework. This process allowed an inter-rater reliability measure to be calculated as outlined by OConnor & Joffe (2020). If the two researchers fell below 80% of consistency of coding then a meeting occurred where the inconsistencies were discussed and resolved. Nine interviews (none of which were coded in Rounds 1 or 2) were checked in this way, with at least 25% of each interviewee group checked. The remaining interviews that were not used in Rounds 1 or 2 were reviewed by one of the other authors, that is, the first author sent them the segments of text they had coded with the codes attached. This process ensured that the results of the coding of any interview were not solely dependent on the first author’s understanding of the data.

Round 3 coding resulted in one new code being added to the framework (‘Benefits-Technology’) and the consistency checks resulted in more precise definitions for many of the Round 2 codes. This process produced version three, V3, the final version of the coding framework.

#### 4.1.4 Round 4: reviewing codes ($n=23$ interviews reviewed)

The coding of all interviews was revised using the final coding framework by the first author, and the results are based on this coding.

#### 4.1.5 Theme identification

Upon completion of the coding process, identification of the themes highlighted by the coding took place. A key objective of this process was to detect evidence that pointed to any differences in the experience of tutors and students in this new online MSS context, while attempting to attend to the potential impact of these experiences on MSS in post-COVID-19 settings, as per the research question. Similarly, attention was paid to any testimony of shared experiences both from a tutor-student perspective and an Australia–Ireland perspective.

### 4.2 Participants

Participants in this study were recruited via email by the second author (for WSU students and tutors) and the third author (for UCD students and tutors) in October 2020. Students from both WSU and UCD were only sent the recruitment email if they had used MESH or MSC services after the transition to online MSS. Those emailed were $n=890$ MESH users and $n=397$ MSC users (231 of which were first-year students who only had access to the online MSC from when they started university in September 2020). All tutors except the authors of this paper in both MESH and the MSC ($n=11$ and $n=15$, respectively) were emailed and invited to participate as they all had tutored online during the relevant period of this study.

As stated, seven WSU students (AS1–AS7), four WSU tutors (AT1–AT4), six UCD students (IS1-IS6) and six UCD tutors (IT1–IT6) agreed to be interviewed. The fact that these are convenience samples has imposed limitations on their analysis. However, the student samples were diverse in makeup based on course, stage, gender and their pathway to university.

The UCD students were a blend of first- and second-year students from both service mathematics courses and specialist mathematics degree courses. One of these students had used the physical in-centre support service prior to COVID-19 and three students were first-year students whose only experience of university was in an online setting. All but one of the UCD tutors were postgraduate students with between three and seven semesters of experience in MSS tutoring, the exception being an experienced MSS tutor who had also lectured in the School of Mathematics and Statistics for several years. The WSU students were five first-year and two second-year students, all studying mathematics as part of a non-mathematics degree. Only one first-year and one second-year student had experienced on-campus MSS. Three of the four WSU tutors have postgraduate qualifications and at least five years’ experience with MSS; the fourth tutor has at least two years’ experience with MSS tutoring. Contrary to the UCD postgraduate tutors, WSU staff would be classified as ‘dedicated staff’ according to the MSS staffing definition of Lawson *et al.* (2020, p. 1238).

## 5 Results

**Fig. 2.**
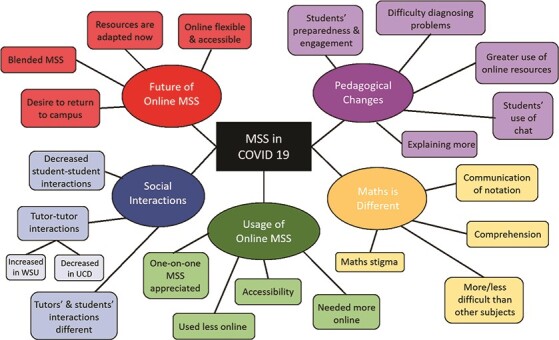
The five themes and their subthemes identified in the analysis of the interviews.

Analysis uncovered five themes as central to the shared experiences of tutors and students in the online MSS context of the COVID-19 period of March to November 2020. These are *Usage of online MSS*, *Mathematics is different*, *Social interaction*, *Pedagogical changes* and the *Future of online MSS*. The themes and their subthemes are shown in Fig. 2. We now focus on the first, second and fifth themes as they relate to the research question. As participants’ views were largely similar regardless of their institution, the common perspective is presented unless otherwise stated.

### 5.1 Usage of online MSS

As discussed, online MSS was utilized significantly less by students in WSU and UCD during 2020 in comparison with in-person MSS offered previously. The theme of how and why online MSS was used by the students arose in the interviews yielding four subthemes (shown in green rectangular nodes in Fig. 2), three of which will now be discussed. Tutors and students commented on this decrease in use and some expanded on why this may have occurred. However, the tutors and students we interviewed confirmed there was a core group of students who really appreciated the support offered online, and some were in fact more likely to use online MSS than in-person support.

#### 5.1.1 Used less online

Tutors commented on how they saw fewer students online, particularly towards the beginning of the wholly online period. One tutor noted online support was ‘a bit quieter’ than the on-campus support despite MESH combining six campuses into one Zoom room facility. They explained the following:

I would work at a specific campus, and I might get three or four students in a session but now even though it was all of the campuses put together, like in one session I might not even get that much. … Maybe the students were a bit apprehensive about whether they could actually get help online. (AT4)

UCD tutors spoke similarly about fewer students accessing support online, although one was hopeful that with the increased number of students traditionally using MSS in the Autumn (September-December), visits would pick up.

In the beginning students were very cautious about the whole thing, in that they didn’t know how it worked. … they would be … less likely to just drop in randomly as they would in the in-person MSC, which I think is probably quite a big disadvantage … it’s just they would be less likely to when they don’t know what they are going into or when they can’t take a look from the outside before they are actually in the room. … But I think this semester things are getting a lot busier and students are used to the online thing … I think the longer it will go on online I think the more they will get used to it and the less sort of hesitant they will be. (IT2)

Although online bookings picked up from the start of the next academic year, MSC visits remained relatively low for semester one of 2020/21 with 1,515 online visits compared to 3,127 in-centre visits in the corresponding 2019 period. While tutors were not asked explicitly for reasons why they thought there was a such a drop in MSS usage, one tutor worried that a certain type of student was no longer utilizing MSS:

we still might not be reaching out to the students that would sort of just drop in, … like they might have in the library because it was there. (IT4)

The students helped provide insight into why online MSS might be less accessed than in-person support. AS1 explained that they turn off their computer when studying and while they would have spoken to MESH tutors frequently in the library while studying there, studying at home meant they would have to turn on the computer to speak to MESH tutors. Even when students do turn on their computer they admit it is easier to use YouTube as it is available 24 hours a day. Another student stated that online one-on-one interactions make them anxious, although they had encouraged their fellow students to use MESH:

Just knowing the support is there is the first thing … when I hear people saying that they’re having trouble with it [mathematics] I will try push them towards MESH or PASS or whatever is available. But I’d guess a lot of people … either have trouble working out that they need help or have trouble telling other people that they need help. (AS4)

A UCD student explained a similar situation among their peers, where despite the extra support set up by the MSC, students still cannot attend due to being overwhelmed or fatigued:

students can be … not lazy, but like when you’re trying to get to all your classes, sometimes it can be hard to go and … get the energy to reach out to the MSC or reach out to the lecturer and be like, “Oh, I don’t get this”. … the lecturers and tutors are making a massive effort to get us to use the supports, but I don’t know if they’re being fully used by students. (IS5)

#### 5.1.2 Needed more online

In contrast to the decreased use of MSS subtheme, several students believed they used MSS more often when online.

AS5, who struggled to connect with their peers online, noted that they probably would have used MESH less if they had study partners to collaborate with:

if I had study partners I potentially would have used MESH less. But without that I needed that additional support to be able to just have that explanation of why I couldn’t figure out the stuff. … But having that human interaction made a big difference.

UCD students IS2 and IS4 found learning mathematics online brought them to the realization that getting support can be a positive action. IS4 said the following:

it kind of made me realise that sometimes you do need to ask for help but it’s not that you are stupid and you don’t get it, it’s just maybe that there is a gap in the knowledge that needs to be filled.

Both students also stated that they would have used MSS less in person as they were struggling more with mathematics online and so took action by accessing the MSC. IS2 explained that they take great pride in figuring out mathematics independently but found this was not manageable when studying from home due to their increased workload. IS4 found that online mathematics learning made them notice knowledge gaps that would have been filled subconsciously in person, in part through peer interaction.

Another UCD student noted that they themselves were equally likely to use either the in-person or online MSS: ‘It’s probably one of the areas that hasn’t changed that much because they adapted really quickly on to online’. However, they also commented that

Some of my friends are more willing to just do it [access MSS] online as opposed to face-to-face because … you just type it in, it’s kind of less, I dunno, as opposed to walking in, I suppose. (IS5)

#### 5.1.3 Accessibility

Online MSS has the potential to be more accessible for some students. AS3 noted the following:

I think that the accessibility makes you feel more like, look I can actually do this. It’s actually more of a possibility. You don’t have that excuse of ‘I have to go into campus’.

AT2 also commented that there was a possibility that the online MESH support might suit students who would not ordinarily have attended in person, whether this is due to timetabling reasons or the safety of greater anonymity. They believe online MSS can attract different personality types. A UCD tutor also thought online support would make it a bit easier for students:

to come to the support centre and be prepared just because it’s kind of easier to there, there’s more flexibility on their part in the times they can do. Before it was, …you have a free hour in between lectures and …it’s kind of the best time to go. And, you can’t go in the evening, because maybe you know you have to catch your bus …(IT5)

However, they still believed that there were similar or even lower levels of student engagement at the MSC than when in person.

### 5.2 Mathematics is different

The move to online MSS has reinforced the idea that learning and tutoring mathematics and statistics is genuinely different to other subjects. While the interview questions anticipated this, the volume of responses to this notion was surprising. These differences include difficulties in communication between students and tutors, comprehending mathematics online, and the extra concentration required to both tutor mathematics from tutors’ perspectives and to absorb content from the students’. Specific disciplines mentioned in comparison to learning and teaching mathematics included commerce, languages, laboratory-based disciplines, medicine, economics and the so-called ‘hard’ discipline of engineering. Strong comparisons were also made between teaching and learning mathematics in person and online. In terms of conceptual understanding, the need for maximum attentiveness when being tutored was put forward as an extra hurdle when learning mathematics online as opposed to the in-person support setting.

#### 5.2.1 More/less difficult than other subjects

The feeling among WSU and UCD tutors was that the move to the online environment has reaffirmed their belief that mathematics is different to learning other subjects and is generally more challenging to tutor and learn. AT3 expressed their desire to discontinue tutoring mathematics online. They discussed how communicating mathematics specifically is slow and frustrating and that those who have not tried to teach mathematics online do not understand how difficult it is. This was based on their experience of delivering MSS exclusively through Zoom where students used the chat facility and therefore had limited capacity to render mathematical symbols. If their job were to be permanently online, they would leave it.^2^

I think if we had to go online forever I definitely would not want to do that and I’d be looking at … doing something else. I think it’s reinforced that mathematics and statistics are different to most other content areas. … It’s made me realise how important communication is. (AT3)

We note that not all tutors were of the opinion that mathematics learning and teaching was harder now that it was online, with one tutor (AT4) stating that mathematics teaching was no different whether in person or online once the initial adaptation period had been overcome. Despite this opinion, the tutor’s response aligned with other tutors interviewed in that the nature of mathematics impacts their ability to tutor effectively online.

Initially, there was agreement among WSU students about whether mathematics is easier or harder to learn online than other subjects. When asked directly six of the seven WSU students said there was nothing specific about mathematics or statistics that made it more or less difficult to learn online. AS2, who studies science, would happily study their mathematics subjects online with their science classes on campus as mathematics was comparatively more amenable to online learning. AS4 noted that while there was initial difficulty in accessing online academic support eventually online communities (e.g., using Discord) grew and became active and as a result mathematics became easier to learn online. AS6 appreciated seeing mathematical problem solutions ‘zoomed up in front of your face’ as they found it easier to focus on what they were being shown.

However, within the broader scope of their full interview, all these students reported at least one aspect of mathematics that made it more difficult to learn online. AS1 upon comparing their current mathematics studies with their previous commerce degree expanded on the difficulties of comprehending mathematical concepts online. They noted that full concentration is needed throughout a mathematical explanation and that missing one step, perhaps as the wi-fi loses connection briefly, can cause extra work.

All six UCD students identified that there were difficulties in learning mathematics online due to an increased need for understanding in comparison to other subjects. IS5, studying both Economics and Mathematics, compared these subjects:

My economics studying hasn’t changed at all, really because they kind of just put up the material, you go through it, you listened to their slides and then you just give the questions a go and you’re grand. Whereas with maths and stats stuff, it’s a lot more like, it’s all about your own understanding. And I feel like it’s become a lot harder to understand something because … they still explain things, but it’s not how they used to … They’re not as … solid as they used to be.

#### 5.2.2 Comprehension

Overall students stated comprehending mathematics on their own without support takes longer online and that complete explanations and concepts are easier to understand in person. Two Irish students spoke about the need to understand the concepts in mathematics which for them is a difficult process. It is not useful to just ‘learn it off’ by heart (IS4) but students have to understand ‘where they [concepts] come from, how they are meant to work and why they work’ (IS2).

Even when students access online MSS, due to the difficulty in communicating mathematics online there is still a barrier to complete understanding as highlighted by IS4:

In maths it’s concepts and sometimes trying to maybe explain the concept you are confused by is difficult enough, let alone if you are trying to do it through a screen. They’re trying to help but … you don’t fully know what you are confused by so they don’t really know how to help you to be less confused.

These difficulties in learning mathematics online have caused two students (IS3 and AS7) to consider deferring future mathematics courses until they are available as in-person courses. IS3 explained throughout their interview that they enjoy ‘hands on maths’ and they found it too difficult to learn mathematics online as this practical feature was significantly decreased. AS7 stated that in-person mathematics teaching is far more engaging and they would also prefer not to be assessed online any longer.

#### 5.2.3 Communication of notation

The physical act of writing and drawing mathematics, both for tutors and students, is hindered in the online MSS environment. As time progressed, it was evident that tutors in particular became more equipped with the requisite technologies (tablet, stylus, etc.) to mitigate these issues but not every student had such luxuries.

In terms of specific content, tutors expressed more difficulty in supporting applied mathematics, when ‘there’s just so much writing to get through’ (IT1), and computer programming or coding online (in particular R and Python) than in person. However, IT6 and AT1 who also gave regular coding tutorials found tutoring coding to a class online easier than in person though the situation was reversed when it came to coding in the one-on-one MSS context. In this setting, the tutor and student need to see (a) the question, (b) the student’s attempt and (c) the tutor’s intervention which ideally requires the same code to be run and displayed on one screen which is easier to do in person when the tutor may work on the student’s machine.

On a more granular level, tutors struggled communicating mathematical notation, language and symbols and annotating student work. In particular, the use of language was a barrier to communication specific to mathematics with one tutor saying the following: ‘Things like they’d be reading out their equation to you and, it’s really difficult to read out mathematics, no one should ever do it.’ (IT1), and another stating the following:

We’ve students trying to type maths into the chat, because that’s the only way they’ve got to communicate and it really makes it much harder than other subjects. (AT3)

A third tutor noted that in addition to students’ difficulty in being unable to write numerical scripts, tutors also felt the loss of being able to check the setting out of students’ work. This was difficult to achieve when annotating virtual whiteboards that got ‘messy’. They summed up their feelings about communicating mathematics online stating the following:

You can talk about it but there is nothing like doing it and seeing it. So I think that was missing a little bit. I found that quite difficult. (AT2)

This sentiment was clearly echoed among all tutors interviewed.

#### 5.2.4 Maths stigma

Another distinguishing feature of mathematics is the widespread acceptance that it is okay to dislike or be unable to learn mathematics. Motivating such students, as reported by tutors, is much harder online than in person where physical gestures and verbal encouragement, based on students’ body language and work, can be more empowering.

without the kind of real physical … facial expressions, say “I believe in you”. Without that it’s definitely a little bit more difficult I would imagine than other subjects, just because the general feeling is “No, I don’t like maths”. (IT3)

One student also referenced stereotypes around mathematics that require addressing whether in person or online:

I’m not sure if it’s more difficult to learn online., but there’s … the whole mental health thing. There is this weird stigma about maths … I think there needs to be a shift there. … but it hasn’t been less difficult, I don’t think. (IS1)

In summary, tutors and students identified many aspects of mathematics learning that are affected by the move online, some positive but mostly negative.

### 5.3 The future of online MSS

With less MSS engagement and difficulties of online learning exacerbated by the nature of mathematics, students and tutors interviewed aspired for the future of MSS to be different to its current pandemic-enforced online state. However, some positives to online MSS have revealed themselves through the forced move and so participants do not wish the future to look exactly like previous on-campus MSS.

#### 5.3.1 Blended MSS

While the majority of tutors can speak to various benefits of online MSS such as greater accessibility afforded by video conferencing compared to traveling to campus (particularly in WSU’s multi-campus structure), none of the tutors interviewed would like MSS to continue in a fully online setting. Most tutors were open to a blended form going forward:

To be honest, I started off being a little afraid of coming online. I think it’s just, cause I’m not used to it, but I found it to be much better than I thought it would be. …maybe not in programming or computers, but in other ways it’s definitely an option. Like if someone were to suggest 50 50 in person or online, there’s certainly a lot of queries that students have that are 100% like you can do them in 10 minutes online kind of thing. (IT1)

The hybrid model seems to be a viable option among WSU tutors with AT1 and AT4 speaking extensively on this. As AT1 explains, it must be designed with students’ needs in mind.

I think it’s good to have that online option always there. I don’t think it’s a good idea to go … even with something like MESH, to go completely online because like I said some students just don’t have the technology to learn and be comfortable with it; but at the same time, for all the students who are comfortable I think it’s definitely good to have that online option there; especially at Western Sydney because we have so many different campuses.

#### 5.3.2 Resources are adapted now

Tutors who have found a way to make online MSS work are hesitant to give it all up again once students and staff are back on campus. Even AT3, who would not continue working as a tutor if MESH was permanently online, would not like to see the online resources they spent many hours creating never used again, ‘I wouldn’t say that I prefer them, but I think they’re valuable.’

#### 5.3.3 Online flexible and accessible

Students vary more widely in their opinion of what type of MSS they would like to receive in the future. Some first-year students interviewed had not experienced on-campus MSS and so were unable to compare. While some expressed dislike for their entire mathematics learning being online, WSU students as a whole were keen to keep online drop-in MSS for its convenience and flexibility:

I would like to keep up with the online interactions, because the days I don’t go on campus, … it will be excellent. (AS1)

#### 5.3.4 Desire to return to campus

The WSU students did however express a desire for some return to on-campus support as that longed for in-person interaction is just not possible by Zoom:

I think the face to face you get more of that, … like somebody will want to explain a little bit more to you because they can see your face and see your interaction. If you don’t get it. … although you can do that in Zoom, but because of the time limitation in Zoom, sometimes it’s just not easy. (AS1)

UCD students were even stronger in their preference for in-centre support, with those who had not experienced it eager to find out what it was like:

And the in-person centre, I haven’t gone there yet. … once I get the chance, I’ll definitely be like in person working. (IS3)

UCD students who had experienced in-centre support were eager to return to campus and leave behind all the technical problems experienced with online MSS:

I suppose most of the time it’s grand, they are there to help you and … explain what you are confused by. But sometimes if you have connectivity issues or they are trying to share their screen but their screen gets frozen … this happens more times than you think; … you are trying to have the question up and then have the screen up and then also maybe share your notes you have written yourself as well and then … it’s a lot more easier to sit down beside someone. You have your page; you look at the page and you write on the page. (IS4)

One student spoke to the psychology of in person versus online support and believes being in person helps in confronting problems—a key element of MSS for them.

I believe that if you confront your problems with people around you, it’s a really good boost to your confidence and individual development. I think that’s important; that people don’t hide behind the screen so much. (IS1)

There is no mistaking tutors’ desire to return to work on campus—to interact fully with students, to be able to use body language and to see all the work clearly in one place instead of swapping between computer screens. As IT2 explains

I think I prefer being able to see someone; as in face to face; and just real time reactions and that sort of thing. I prefer working with a pen and paper just because it’s faster first of all and because you can bring emphasis to parts of it a lot easier. (IT2)

While the majority of tutors are now comfortable with working online and are ready and willing to continue with online work in some fashion, on-campus support is preferable. As AT3 notes, when online they do not get that buzz from doing it like person to person.

In general, there is an acceptance that online MSS can be effective in certain situations where the student is comfortable working in that environment. However, the strong desire to be in person, and hence back on campus, is evident among the study’s participants.

## 6 Discussion

This study has brought to light a range of issues related to the provision of online MSS in the COVID-19 era. The themes outlined in the results section, concerning MSS usage patterns, the distinguished nature of mathematics as a difficult discipline to learn and support online and attitudes to how support provision might be re-imagined in a post-COVID world, stand apart but are linked. Emergent questions about the relationship between students’ use of mathematics learning support and their perceptions of the value of online learning in this discipline could be explored further in research with a narrower focus than the present scoping study. Similarly, the perceptions of students who have only experienced online study of the challenges and opportunities of studying mathematics online could be compared to the perceptions of students who have received some in-person instruction.

While many of the issues raised in this study have been accounted for in a large corpus of online higher educational literature, it must be emphasized that this study foregrounds the experience of students and tutors of mathematics engaging with their study and practice during the COVID-19 pandemic. It is interesting that many of the themes in related studies that predate the pandemic resonate with those that have emerged in this study. This fact should pose questions for practitioners and researchers of MSS, and also senior managers, about the implications of the fact that mathematics is different (Smith *et al.*, 2008; Trenholm, 2013; Trenholm *et al.*, 2019), what can be done about reduced usage in online support provision and in what ways support services should be remodeled for post-COVID delivery.

The theme addressing students’ and tutors’ use of online MSS raises a number of questions. The fact that usage has significantly decreased at WSU and UCD (as reported in Section 3 and as seen in Hodds, 2020 and Johns & Mills, 2021) suggests challenges for support providers related to accessibility, use and availability of technology, staffing and advertising. Reasons for the decrease cited by the interviewees span problems in adjusting to online learning platforms and media, the perceived anxiety-inducing nature of online support when it comes to the intensity of one-on-one interaction in a confined digital environment and fatigue or depleted motivation due to the ‘overwhelming’ experience of studying online during the pandemic. Some students studying off-campus found it easier to search the web for resources than to join an online session or make an appointment, whereas on campus it was easier to use drop-in support.

An interesting contrast in the student responses was that while support usage dropped, those who used the online services available to them valued it at least as much as the in-person offerings available to them at earlier times. This could be due to an increased need among students for support in study routines affected by isolation (from peers and tutors) and less opportunity for incidental ‘corridor’ discussion. Two UCD students stated that they used MSS more online than in person; however, it was the lack of interaction with others that was behind the desire for extra assistance and this overrode the disadvantages of online MSS outlined in Section 5.2.

The theme highlighting aspects of online mathematics teaching, learning and support that mark it as different from other disciplines emerged from the interview responses as a compelling testament to something the authors, as experienced mathematics educators and researchers, have long been aware of in their own practice: the technologies and environments used for online mathematics learning can enable but also impede such things as effective written, oral and non-verbal communication, corrective feedback mechanisms, modelling of methods and solutions, use of visual-spatial explanations and instructional efficiency. The expanded discussions of the discipline-specific characteristics of mathematics given in Trenholm *et al.* (2019), Protopsaltis & Baumi (2019), Trenholm (2013), Paechter & Maier (2010) and Smith *et al.* (2008) reinforce these ideas and highlight the need for research in this space that is centred in the discipline.

With respect to online communication of mathematical concepts and methods, there was a consensus view among the interviewees that this was complicated by the idiosyncratic nature of the discipline. The problem of how to induct students into use of technology to write mathematical symbols and organize text according to strict layout conventions was raised, alongside the perceived awkwardness of out-loud readings of mathematical phenomena (such as equations). In addition to the limitations encountered in notating, mathematical language was the problem of easily conveying meaning in the language itself, primarily due to the fact that crude platforms (such as Zoom chat) were being used clumsily in situations that, were the exchanges to happen in person, might be dealt with by a swift verbal or written interaction. These issues, whose effect has been to hinder teaching and learning, are well documented and serve as a reminder that to date no universally accepted and effective online substitute has been found for in-person communication of mathematics (Meehan & Howard, 2020; Trenholm *et al.*, 2019).

These challenges had follow-on effects in disabling many tutors’ basic pedagogical functions such as checking the set out of students’ work and stepping them through explanations in a more assured manner than was possible in transient, digitally mediated spaces. In many cases, tutors had to adjust their practice to compensate for faces that were not seen and voices that were not heard (Trenholm, 2013). A problem of space arose here, with tutors reporting the difficulty of juggling multiple interfaces (virtual whiteboards, browsers, software applications, lesson worksheets, etc.) at the same time in order simply to establish a display for demonstration and discussion with students. A possible implication of this is that mathematics tutors, due to feelings of frustration, incapacity or restraint, develop negative attitudes to working in online environments and contemplate quitting or suffer a style of disillusionment that impacts the quality of their tutoring. There is substantial evidence in this study that tutors and students recognize the benefits of operating online, so the task for practitioners and management is to be honest about the above-mentioned disabling factors in plotting a way forward that solidifies these benefits but minimizes the challenges. ‘Best practice’ guides for online MSS will no doubt be useful in this endeavour (Johns & Mills, 2021).

Perhaps the most consequential finding to emerge within the ‘mathematics is different’ theme was that, due to its conceptual and sequential nature, mathematics demanded special attention from, and placed special cognitive stresses upon, students and tutors. According to the student interviewees, this put it at odds with other disciplines and rendered it more difficult to learn in situations absent of human co-presence, a sense of community (among peers, for example) and the opportunity for ‘short-cycle’ instruction (Trenholm *et al.*, 2016). In fact, as far as some students (and two tutors) were concerned, this was sufficient reason for them to avoid studying (or tutoring) mathematics subjects online, if such a choice were open to them. This study makes no claim about any *objective* measures of difference between the conceptual demands of mathematics compared to other disciplines, but it is noteworthy that this issue appeared so prominently in the response data—perhaps indicating a widely held *perception* or stigma that has tangible effects on the engagement behaviours of students and tutors.

Considering the fact that, with the move online, the MSS tutors interviewed lost physical presenting space, the ability to see students’ work and communicate in subtle ways using body language, it was not surprising to learn of their strong desire to return to campus. Most students, struggling with the more independent nature of online learning, as well as pandemic-enforced isolation, expressed a similar desire. This affirms reporting in the pre-pandemic literature of students’ preferences for in-person mathematics study (Jaggars, 2014; Xu & Jaggars, 2014). Unfortunately, at the time of writing, such a return is not possible at UCD and in only limited ways at WSU. The opinions of students and tutors reported here can inform future MSS plans. Students’ perception that they cannot teach themselves mathematics (Jaggars, 2014; Trenholm, 2013) is a problem that needs further, and urgent, research as many universities recalibrate their learning support programs in response to the pressures of COVID-19. Exploration of the struggles of tutors in adjusting their in-person pedagogy for online use is also needed, perhaps framed by the contestable notion of the preeminence of pedagogy over medium (Jaggars, 2014; Trenholm, 2013).

The benefits of online MSS that have been identified here—tutors and students appreciating its flexibility, students liking its affordance of anonymity and tutors valuing its provision of new learning resources—must be considered when deciding how to deliver online MSS in the post-pandemic world. Wider evidence of these benefits should also be factored in, but with an awareness of the presence in the literature of over-hyped commentary (Danielson *et al.*, 2014; Figlio, 2016; Jaggars, 2014; Protopsaltis & Baumi, 2019; Trenholm, 2013; Trenholm *et al.*, 2016). With regard to students’ attitudes towards post-pandemic use of online MSS, there were some situations identified as amenable to support in this setting. Once students become comfortable with the online environment, it is possible that MSS usage will increase overall if both on-campus and online options are made available and are seen as attractive. In any blended offering, however, it is clear that students and tutors would prefer in-person support to have precedence.

In the short term, those responsible for planning and delivering MSS must acknowledge the issues affecting online support raised in this study and, where possible, try to find solutions. Why students are using MSS less in the online versus in-person mode is still unclear and is a fertile topic for further investigation. Despite possibly new cohorts of students being attracted to MSS because of its online options, the overall decline in users indicates that there are many students going without much needed support (a fact which could impact measures of success and retention). The students and tutors featured in this study have adapted reasonably well to the challenges of communicating online but the fact that it is a slower and more difficult process has planning implications: how much time and how many resources should be given to online MSS?

## 7 Conclusion

The benefits and challenges of delivering and receiving online MSS in the COVID-19 era under the themes of *Usage of online MSS*, *Mathematics is different* and *The future of online MSS* highlighted in this study indicate how much MSS provision has changed and will continue to evolve. More research into why students are not using online as much as on-campus MSS, and ways to improve online communication, is needed to help MSS practitioners and researchers to understand, adapt to and perhaps capitalize upon this change. Moreover, this study did not focus on the experiences of students unwilling or unable to engage in synchronous MSS sessions, a key piece of the puzzle in gaining a fuller picture of online MSS engagement.

While each MSS provision has its own requirements and demands, there is definitely commonality when it comes to meeting the needs of students and ensuring student satisfaction with the service. These needs have been exacerbated by the COVID-19 pandemic due, in part, to the special nature of mathematics and the difficulty of tutoring and learning mathematics online. The significant decrease in student usage of MSS has implications for the future of online MSS. There is a strong desire to return to in-centre support, but with online services continuing in some capacity, showing how the pandemic-enforced move to online learning has permanently changed MSS.

## Footnotes

1 In this paper, ‘subject’ refers to what is sometimes called ‘unit’ in Australia and ‘module’ in Ireland where both terms refer to a standard unit of an instructional section within a university program that is a ‘self-contained’ component of instruction.

2 We note that an (interviewed) Irish tutor ceased working in MSS from January 2021 due to the move to online tutoring stating they had lost too much connection with both their students and fellow tutors.

## APPENDIX

### A. Interview Questions

#### A.1 Student questions

1. Could you tell me a little about your experience of online learning/teaching mathematics since last March?
  (a) What were the challenges?
  (b) What were the benefits?
  (c) What are the main differences for you between online and face-to-face learning/teaching?
2. Is there anything about the subject of maths specifically that makes it more or less difficult to learn/teach online as compared to other subjects?
3. Turning now to your experience of receiving/providing support online in your maths and stats study, how would you describe the experience?
  (a) How frequently have you used/provided support services?
  (b) What type of services have you used/provided?
  (c) Has the way you access/provide support changed since COVID-19?
  (d) If so, why?
  (e) Have you received support in any subjects other than maths or stats?
  (f) If so, how does the support you have received in maths and stats compare to the support you have received in these other subjects?
  (g) Do you usually access support individually or in a group?
  (h) Has the support made you feel more connected to your fellow students, teachers or the university more generally?
  (i) Where else have you gone for help (if not a dedicated learning support service)?
4. Tell us about your interactions with support staff online:
  (a) Is it easy or difficult to communicate with them?
  (b) Do they make you feel comfortable and ready to learn?
  (c) Are they usually successful in helping you with your problems or questions?
5. If you have received both online and face-to-face support in maths and stats, could you tell me about the differences between the two?
  (a) When you seek help from a teacher because you don’t understand a maths concept or solution method, for example, is it easier or more difficult to discuss your issues with them online or face-to-face?
  (b) Do you interact with other students differently online vs face-to-face?
  (c) Are you more comfortable receiving support online vs face-to-face?
  (d) Are you more confident in seeking help online vs face-to-face?
6. Has receiving support online changed your attitude to the subject in any way?
  (a) Has that change, if any, been positive or negative?
7. If your future subject choices were flexible, would you wish to study more or less university maths in the future?
8. Are there any elements of online support that you prefer and would not want to lose upon resumption of ”normal” arrangements (post COVID-19)?
9. What about the opposite: are there any elements you do not prefer?

#### A.2 Tutor questions

1. Could you tell me a little about your experience of online teaching mathematics since last March?
  (a) What were the challenges?
  (b) What were the benefits?
  (c) What are the main differences for you between online and face-to-face teaching?
2. Is there anything about the subject of maths specifically that makes it more or less difficult to teach online as compared to other subjects?
3. Turning now to your experience of providing maths and statistics support online, how would you describe the experience?
  (a) How frequently were or are you providing support services?
  (b) What type of services have you provided?
  (c) Has the way you provide support changed since COVID-19?
  (d) If so, why?
  (e) How would you describe your interactions with your fellow teachers online?
4. Tell us about your interaction with support users (students) online:
  (a) Is it easy or difficult to communicate with them?
  (b) Are you usually successful in helping them with their problems or questions?
5. If you have provided both online and face-to-face support in maths and stats, could you tell me about the differences between the two?
  (a) When you are speaking to a student, is it easier or more difficult to discuss their issues with them online or face-to-face?
  (b) Do you interact with other teachers differently online vs face-to-face?
  (c) Are you more comfortable giving support online vs face-to-face?
  (d) Are you more confident in giving help online vs face-to-face?
6. Has giving support online changed your attitude to the subject in any way?
  (a) Has that change, if any, been positive or negative?
7. Has this affected how you think about your future in teaching maths and stats?
8. Are there any elements of online support that you prefer and would not want to lose upon resumption of ”normal” arrangements (post COVID-19)?
9. What about the opposite: are there any elements you do not prefer?

### B. Bios and contact details

**Claire Mullen** (claire.mullen@ucdconnect.ie) is a PhD student in the School of Mathematics and Statistics at University College Dublin (UCD). Her research interests include third-level mathematics education with a focus on mathematics and statistics support. Claire has tutored at the UCD Maths Support Centre for over two years and received a BSc (Hons) in Mathematics, Applied Mathematics and Education from UCD in 2020.

**Jim Pettigrew** (j.pettigrew@westernsydney.edu.au) is a lecturer within the Mathematics Education Support Hub at Western Sydney University. His research encompasses university mathematics and statistics learning support, nursing numeracy, test design and analysis and equity in mathematics education. Prior to working at Western Sydney University, he received a PhD (mathematics) from the University of NSW in 2009, a Diploma of Education from the University of Melbourne in 2003, a Masters (mathematics) from La Trobe University in 2001 and a Bachelor of Arts (Hons, mathematics) from La Trobe University in 1998.

**Anthony Cronin** (anthony.cronin@ucd.ie) is a lecturer and Mathematics Support Centre manager in the School of Mathematics and Statistics at University College Dublin. His research interests are in the spectra of non-negative matrices and tertiary mathematics education. Anthony received the UCD President’s Teaching Excellence Award in 2021 and the University Award for Outstanding Contribution to Student Learning in 2017.

**Leanne Rylands** (l.rylands@westernsydney.edu.au) is an associate professor at Western Sydney University, Australia. Leanne is the head of the Mathematics Education Support Hub and a member of the Centre for Research in Mathematics and Data Science. Her research interests are in tertiary mathematics education and combinatorics. Leanne received a national teaching award in 2010 for outstanding contributions to student learning.

**Don Shearman** (d.shearman@westernsydney.edu.au) is a learning adviser of the Mathematics Education Support Hub and sessional academic in the School of Computing, Data and Mathematical Sciences at Western Sydney University. He has over 30 years experience in teaching and supporting mathematics education. His research interests focus on the effectiveness of mathematics education support. Don's BSc (Hons) and MSc both in pure mathematics are from the University of Sydney and were earned too long ago to remember the years.
